# Treatment of Richter’s Transformation with Novel Therapies

**DOI:** 10.1007/s11899-023-00721-8

**Published:** 2024-01-09

**Authors:** Amneet Bajwa, Alma Habib, Adam S. Kittai

**Affiliations:** The Ohio State University, 2121 Kenney Road, Columbus, OH 43210 USA

**Keywords:** Richter’s transformation, Richter’s syndrome, Therapy, Chronic lymphocytic leukemia, Novel therapies

## Abstract

**Purpose of Review:**

This review presents recently published clinical trial data and ongoing investigations regarding the treatment of Richter’s transformation (RT).

**Recent Findings:**

Recently, numerous approaches have been investigated for the treatment of RT including: traditional chemoimmunotherapy regimens combined with targeted agents such as BTKi and BCL2i; immunotherapy combined with targeted agents; non-covalent BTKis; bispecific T cell engagers; and CART therapy. In addition, various novel targeted agents are currently being studied for the treatment of RT in phase 1 and 2 clinical trials.

**Summary:**

Standard of care treatment with chemoimmunotherapy for RT has limited efficacy in achieving durable remissions. Here, we review recent data on the use of combination treatments and targeted agents in RT. Although some progress has been made in the investigation to optimize treatment of RT, further study is needed to evaluate long term outcomes of recently published trials and test efficacy of upcoming novel agents.

## Introduction

Richter’s transformation (RT) refers to the transformation of chronic lymphocytic leukemia (CLL) into an aggressive lymphoma and is associated with poor clinical outcomes [1]. RT frequently presents as a variant of diffuse large B cell lymphoma (DLBCL-RT) in 90–95% of cases and Hodgkin lymphoma (HL-RT) in about 5% of cases [2]. Other rare histological subtypes including acute B-cell lymphoblastic lymphoma and mantle cell lymphoma have been reported [3, 4]. RT can present with symptoms of rapidly progressive lymphadenopathy, sudden increase in lactate dehydrogenase (LDH), and weight loss [2]. The annual incidence rate of transformation to RT is approximately 0.5–1% in patients diagnosed with CLL [5]. In this review, we will provide a brief introduction to RT and discuss the evolving landscape of its treatment.

## Pathogenesis

The pathogenesis of transformation from CLL to RT remains poorly understood. Proposed mechanisms include a potential trigger by a viral infection, such as Epstein-Barr virus (EBV), or accumulation of genetic mutations leading to a more aggressive form of disease [6, 7]. The transformation often arises from the original CLL clone as evidenced by an identical immunoglobulin heavy-chain variable region (IGHV) rearrangement (termed clonally related RT); however, less frequently, it may represent a secondary unrelated lymphoma [1, 8]. Through whole-exome and genome sequencing, clonally unrelated RT has been found to be a distinct de novo DLBCL, lacking shared genetic makeup with the underlying CLL [6, 9•]. In about 90% of DLBCL-RT cases, somatic gene mutations present include *TP53*, c-*MYC*, *CDKN2A*, and *NOTCH1* [6, 8, 10]. These genes play important roles in apoptosis, cell cycle regulation, and cellular metabolism [10]. Little is known of the genetic makeup of HL-RT, although most cases are thought to be clonally unrelated to the underlying CLL and develop as true, de novo secondary malignancies [8].

## Risk of RT Development

Predictors of RT development have evolved over the years. In 2008, Rossi et al. identified biological and clinical risk factors for RT [11]. In a cohort of 185 untreated patients with CLL, CD38 expression, lymph node size (≥ 3 cm), presence of del13q14, and the *IGHV4-39 subset* were risk factors for RT development [11]. The use of 2-deoxy-2-[^18^F]fluoroglucose/positron emission tomography (FDG/PET) and identifying SUV_max_ cutoff of ≥5 of lymph node avidity have been recognized as a useful diagnostic tool to determine whether a biopsy to diagnose RT is needed [12]. These characteristics were historically used to identify patients who were at risk of transformation and help guide patient counseling and management.

With the introduction and widespread use of small molecule inhibitors in the treatment of CLL, clinical and molecular prognostic features of RT have been an important area of focus as it is unclear if our previously developed tools to measure risk of and to identify RT are still valid with these new therapies. Recently, we assessed patients with CLL treated with ibrutinib, a Bruton tyrosine kinase inhibitor (BTKi), who progressed, to determine which clinical features were associated with RT development at progression. We found that lymphadenopathy without lymphocytosis, elevated LDH, and progression on treatment for CLL were associated with development of RT at progression [13•]. Additionally, we reported that the median SUV_max_ for patients who developed RT was 15.2 versus a median of 7.7 for those that had progressive CLL without RT [13•]. This suggests that the traditional cutoff of SUV ≥ 5 to consider biopsy for RT exclusion continues to be a valid marker. Our group has additionally reported the presence of tetraploidy on stimulated karyotypes as highly associated with RT development in patients treated with ibrutinib [14]. More studies must be completed to determine the risk of RT and diagnostic criteria to consider a workup of RT, with other novel therapies such as venetoclax, a B-cell lymphoma 2 inhibitor (BCL2i). Given that chemoimmunotherapy (CIT) is no longer considered standard of care for patients with CLL, it is unclear how these novel small molecule inhibitors might play a role in RT development and outcomes.

## Management

To date, RT has been a challenge to treat with current standard of care not leading to durable remissions [15]. Although patients with clonally unrelated disease have improved outcomes compared to patients with clonally related disease, testing for clonal relationship is not ubiquitous; therefore, we recommend prioritizing treatment of RT on clinical trial regardless of clonal relation as outcomes with RT overall remain poor. The current National Comprehensive Cancer Network Clinical Practice Guidelines® (NCCN) also recommend enrollment in a clinical trial as first-line treatment for RT, although it is preferred for clonally related disease, and only an option for clonally unrelated disease [16]. Prioritizing clinical trial enrollment is also recommended by recent published reviews in the literature [17], as it is essential in broadening our understanding and therapeutic management of RT with the goal of improving patient outcomes. Where clinical trials are unavailable, regimens such as R-CHOP (rituximab, cyclophosphamide, doxorubicin, vincristine, prednisone) and dose-adjusted EPOCH-R (etoposide, prednisone, vincristine, cyclophosphamide, doxorubicin, rituximab) are recommended. If sensitive to first-line CIT, hematopoietic stem cell transplantation can be utilized as a treatment option for additional therapy; however, if the RT is CIT-refractory, a clinical trial should be highly considered, or treatment with regimens recommended for relapsed DLBCL can be deployed. If unable to receive CIT, nivolumab or pembrolizumab with or without ibrutinib can be considered [16].

## Survival

In 2006, prior to the development of small molecule inhibitors, Tsimberidou et al. developed a scoring system to better predict survival of patients with RT [1]. The score is comprised of five adverse factors independently predicting shorter survival in RT [1]. The factors include Eastern Cooperative Oncology Group (ECOG) performance status >1, LDH >1.5x the upper limit of normal, platelet count < 100,000K/uL, tumor size ≥ 5 cm, and more than one prior therapy for CLL [1]. As a prognostic tool, this score accounts only for clinical features of RT; therefore, more advanced molecular predictors of transformation to RT, including *IGHV4-39 subset* and complex karyotype, were studied [11, 18].

The median overall survival (OS) of RT is poor, ranging between 8 and 10 months [19, 20]. Clonally related DLBCL-RT continues to be the most prognostically significant factor, as these patients have a significantly shorter OS than clonally unrelated RT (median, 14.2 months vs 62.5 months, *p* = 0.017) [6, 9•, 21]. In addition to clonal relationship, other clinical and molecular features have proven to be prognostic of survival for patients with RT. We previously identified complex karyotype, defined as having ≥ 3 abnormalities, as a significant adverse prognostic factor with shorter 1-year progression free survival (PFS) and OS of 11% (95% CI: 3–25%) and 18% (95% CI: 7–34%), respectively, compared to 57% (95% CI: 28–78%) and 71% (95% CI: 41–88%) without a complex karyotype [18]. Interestingly, prior treatment of CLL appears to play an important role in RT survival, where the median OS was 7.8 months in patients with prior CLL treatment and 46.3 months in patients with treatment-naïve CLL who develop RT in a large retrospective study [2]. A proposed theory for poorer survival in previously treated patients is the potential for the disease to undergo clonal evolution and develop driver gene mutations in the setting of therapy [22]. Despite the development of small molecule inhibitors for CLL leading to improved outcomes over CIT, DLBCL-RT continues to have poor outcomes [2, 19]. In DLBCL-RT, a phase 2 trial evaluating efficacy and tolerability of R-CHOP in RT showed a PFS of 10 months and OS of 21 months [15]. With EPOCH-R treatment, the PFS and OS were 3.5 months and 5.9 months, respectively [18]. Of note, outcomes of HL-RT are improved in comparison to the DLBCL-RT variant. A recent study showed that patients who received standard ABVD (doxorubicin, bleomycin, vinblastine, dacarbazine) regimen for HL-RT had a median OS of 13.2 years [23].

The emergence of novel therapeutic approaches such as treatment with targeted agents combined with CIT, non-covalent BTKi, PI3K inhibitors, immune checkpoint inhibitors, bispecific T-cell engagers (BiTE), chimeric antigen receptor T-cell (CART) therapy, and other newly developed small molecule inhibitors have the potential to change the trajectory in the treatment of RT. Herein, we will discuss the published data regarding these treatment approaches (Table 1) and highlight ongoing clinical trials (Table 2) which will, hopefully, shed light on optimization of RT treatment.

**Table 1:** Published clinical trials in RT

Identifier	Phase	Author	Intervention	ORR %	PFS (months)	OS (months)
NCT02343120 NCT02795182	1/2 1/2	Tam et al., 2023	Zanubrutinib Zanubrutinib + tislelizumab	61.5% 42.9%	17.3 2.9	29.3 15.4
NCT03054896	2	Davids et al., 2022	VR-EPOCH	62%	10.1	19.6
NCT03054896	2	Davids et al., 2023	VR-CHOP	68%	7.2	19.5
NCT03740529	1/2	Wierda et al., 2022	Pirtobrutinib	54%	NR	13.1
NCT02329847	1/2a	Younes et al., 2019	Ibrutinib + nivolumab	65%	5	10.3
NCT02420912	2	Jain et al., 2023	Ibrutinib + nivolumab	42%	NR	25
NCT04082897	2	Montillo et al., 2023	Atezolizumab + venetoclax + obinutuzumab	68%	NR	31.6
NCT03113695	1	Heyman et al., 2022	Obinutuzumab + HDMP + lenalidomide	43%	5	17
NCT03534323	1/2	Ryan et al., 2022	Venetoclax + duvelisib	37.5	NR	NR
NCT03884998	1	Shouse et al., 2022	Copanlisib + nivolumab	27%	NR	NR
NCT03931642	2	Guize et al., 2022	RCHOP + blinatumomab	46%	NR	NR
NCT04623541	1b/2	Kater et al., 2022	Epcoritamab	60%	NR	NR
NCT03075696	1/2	Carlo-Stella et al., 2023	Glofitamab	63.6%	NR	NR
NCT03144583	1	Ortiz-Maldonado et al., 2022	ARI-0001 CART19	80%	NR	NR
NCT01865617	1/2	Turtle et al., 2017	JCAR014	60%	NR	NR

*VR-EPOCH* Venetoclax, rituximab, etoposide, prednisone, vincristine, cyclophosphamide, doxorubicin. *VR-CHOP* venetoclax, rituximab, cyclophosphamide, doxorubicin, vincristine, prednisone. *HDMP* high-dose methylprednisolone. *NR* not reported

**Table 2:** Ongoing clinical trials in RT

Identifier	Phase	Date Opened	Status	Intervention
NCT03054896	2	02/16/2017	Recruiting	Venetoclax + EPOCH-R or venetoclax + R-CHOP
NCT03899337	2	07/23/2019	Recruiting	CHOP-R +/- acalabrutinib
NCT04679012	2	12/22/2020	Recruiting	Polatuzumab-V + chemotherapy
NCT05172700		12/29/2021	Expanded access	Pirtobrutinib
NCT04781855	1	03/04/2021	Recruiting	Ipilimumab, ibrutinib + nivolumab
NCT04082897	2	09/10/2019	Recruiting	Obinutuzumab, atezolizumab + venetoclax
NCT03534323	1/2	05/23/2018	Recruiting	Duvelisib + venetoclax
NCT03884998	1	03/21/2019	Recruiting	Copanlisib + nivolumab
NCT04806035	1	03/19/2021	Recruiting	TG-1801 +/- ublituximab
NCT04623541	1/2	11/10/2020	Recruiting	Epcoritamab
NCT05207670	2	01/26/2022	Recruiting	Mosunetuzumab
NCT04806035	1	03/19/2021	Recruiting	TG-1801 +/- ublituximab
NCT05873712	2	07/01/2023	Recruiting	Zanubrutinib + lisocabtagene maraleucel
NCT05672173	2	06/02/2023	Recruiting	Ibrutinib + lisocabtagene maraleucel + nivolumab
NCT05537766	2	11/01/2022	Recruiting	Brexucabtagene autoleucel
NCT03833180	1	02/06/2019	Active, not recruiting	Zilovertamab vedotin
NCT04305444	2	03/12/2020	Recruiting	DTRM-555
NCT05107674	1	11/04/2021	Recruiting	NX-1607
NCT05665530	1	12/27/2022	Recruiting	PRT2527
NCT04978779	1	07/27/2021	Recruiting	VIP152
NCT04771572	1	02/25/2021	Recruiting	LP-118

## Targeted Agents and Chemoimmunotherapy

### Covalent BTK Inhibition With and Without Chemoimmunotherapy

Covalent BTKi (cBTKi), such as ibrutinib, acalabrutinib, and zanubrutinib, have revolutionized the treatment of CLL. However, in patients with RT, single-agent cBTKi has not typically led to durable responses. A retrospective review of 4 patients with RT who were treated with ibrutinib notes that 3 patients had a response—1 CR and 2 partial responses (PR); however, the median duration of response (DOR) was only 6.1 months [24]. A phase 1/2 study of acalabrutinib monotherapy in 25 patients with RT demonstrated an ORR of 40% with a median DOR of 6.2 months [25]. One cBTKi which has demonstrated reasonable response in RT patients is zanubrutinib. The CLL-RT1 trial included an arm in which 13 patients with RT were treated with zanubrutinib monotherapy [26]. Results were promising in this arm with an observed ORR of 61.5%, median DOR of 25.4 month and median OS 29.3 months [26]. Although previous studies have demonstrated limited efficacy of ibrutinib and acalabrutinib as monotherapy for RT, this study shows favorable results suggesting that monotherapy with cBTKi may still play a role in the treatment of RT.

The STELLAR trial is a phase 2 randomized control trial investigating R-CHOP versus R-CHOP plus acalabrutinib followed by acalabrutinib maintenance for patients with treatment-naïve RT. The study will also contain two single arm experimental cohorts: The first cohort offers acalabrutinib monotherapy to patients with RT who have progression after CIT as front-line therapy; the second cohort offers R-CHOP plus acalabrutinib to those patients who developed RT while on ibrutinib [27]. Results will hopefully illuminate if cBTKi will have a future role in the treatment of RT when combined with a CIT backbone.

### BCL2 Inhibition With and Without Chemoimmunotherapy

Treatment with venetoclax and an anti-CD20 monoclonal antibody has been shown to result in an improved PFS and deep remission by undetectable minimal residual disease (uMRD) status when compared to standard CIT regimens in CLL [28–32]. Although it has become part of standard frontline treatment in CLL, venetoclax has shown limited therapeutic benefit in RT when used as monotherapy as evidenced by a phase 1 study of venetoclax in patients with R/R DLBCL [33]. Seven patients were included as part of a DLBCL-RT cohort and only 3 patients demonstrated a response. Venetoclax has now been combined with EPOCH-R and R-CHOP as part of a single arm phase 2 study [34]. In this study, 26 patients with DLBCL-RT were treated with 1 cycle of EPOCH-R followed by an accelerated venetoclax ramp up to 400 mg after count recovery. VR-EPOCH was then given for 5 more cycles followed by venetoclax maintenance if response was noted. Responses were notable, with 50% of patients achieving a complete response (CR), of which 11 had uMRD in the bone marrow. ORR, PFS, and OS were 62%, 10.1 months, and 19.6 months respectively. However, there was a high rate of adverse events observed with 65% of patients having a ≥ grade 3 neutropenia, 62% grade 3 anemia, and 50% ≥ grade 3 thrombocytopenia [34]. This led investigators to alter the study and investigate combination venetoclax and R-CHOP. Initial results from an additional cohort investigating venetoclax in combination with R-CHOP for fit patients demonstrated ORR of 68% (*N* = 25) and CR rate of 48%. Median PFS was 7.2 months and median OS was 19.5 months [35••].

### Anti-79b Antibody Drug Conjugate and Chemoimmunotherapy

An additional clinical trial is combining a dose adjusted R-EPCH with polatuzumab-vedotin, an anti-CD79b antibody drug conjugate commonly used in DLBCL, for treatment in patients with RT (NCT04679012). A prior study indicates that CD79b expression is likely to be present in tissue samples from patients with RT and, as a result, may provide a therapeutic target for regimens containing polatuzumab-vedotin [36].

### Non-covalent BTKi

Non-covalent BTK inhibitors (ncBTKi) have been shown to induce responses in patients with B-cell malignancies treated with prior cBTKi [37]. These next generation BTKi have different binding sites than cBTKi and, as result, are effective in treating patients who have previously been treated with cBTKi [37, 38]. In an arm of the phase 1/2 BRUIN study, 50 patients with RT received pirtobrutinib monotherapy, and an ORR of 54% (95% CI, 39–68) was observed [39]. The median DOR was noted as 8.6 months (95% CI, 1.9–NE), and OS was 13.1 months (95% CI, 7.1–NE). In all patients who received pirtobrutinib, (including for indications other than RT), low rates of grade ≥ 3 adverse events were observed with only 15 (2%) patients discontinuing due to a treatment related adverse event suggesting a tolerable side effect profile [39]. Given the ORR and DOR observed, pirtobrutinib might be the ideal drug to bridge patients to more aggressive therapies like anti-CD19 chimeric antigen receptor T-cell therapy (CART) or allogeneic stem cell transplantation.

Nemtabrutinib, formerly ARQ531, is another ncBTKi. The BELLWAVE-001 study included a cohort of six patients with RT who had received ≥ 1 prior therapy [40]. Phase 1 results demonstrated that 25% (10 patients) achieved a PR including 1 patient with RT. Whether nemtabrutinib will have similar outcomes to pirtobrutinib is yet to be determined.

## Combination Treatments with Immunotherapy

### BTK Inhibition and Immunotherapy

To date, there have been two trials which have studied the combination of anti-PD-1 monoclonal antibody (mAb), nivolumab, plus ibrutinib in RT [41, 42]. The first was an open label, phase 1/2a international study which enrolled 20 patients with RT as part of the dose expansion phase of the trial [41]. Notably, patients who had previously received ibrutinib were excluded. ORR was 65% in the RT cohort, and after a median follow-up of 8.7 months, median PFS was modest at 5.0 months (95% CI 2.4–NE), and median OS was 10.3 months. A second phase 2 trial was conducted studying the efficacy of nivolumab plus ibrutinib in 24 patients with RT which demonstrated an ORR of 42% at a median of 28 days from initiation of therapy (range 25–85 days) [42]. Of the 10 patients who responded, 8 had a complete metabolic response on PET imaging, while 2 had a partial metabolic response. Median OS was 25 months for responders as compared to 7.6 months for non-responders [42]. An additional phase 1 trial is ongoing studying the combination of ipilimumab with either ibrutinib alone or as triplet therapy with nivolumab (NCT04781855).

In addition to the monotherapy with zanubrutinib, the CLL-RT1 trial also had an additional arm evaluating the combination of zanubrutinib plus the PD-1 inhibitor, tislelizumab, for treatment of patients with RT [26]. The study enrolled 7 patients in the combination treatment arm. An ORR of 42.9% was observed. Interestingly, the median DOR (25.4 months vs 17.2 months) and median OS (29.3 months vs 15.4 months) were both longer in the monotherapy arm compared to the combination arm. The study did note that the combination arm had a higher percentage of patients with refractory RT as compared to monotherapy arm (100% vs 61.5%), which likely explains the difference in outcomes observed [43]. Based on these results, treatment of RT with BTKi and immune checkpoint blockade does appear to induce a response in a portion of patients, though the duration of the response appears variable.

### BCL-2 Inhibition and Immunotherapy

Recently, results from the MOLTO trial were presented at the 2023 American Society of Clinical Oncology Annual Meeting [44••]. In this multicenter international phase 2 study, patients received a combination of atezolizumab (anti-PD-L1), venetoclax, and the anti-CD20 monoclonal antibody obinutuzumab in untreated RT. In 25 evaluable patients, the ORR was noted to be 67.9%, and CR rate was 28.6%. Median DOR was 11.7 months, and OS was 31.6 months. Treatment-related adverse events were mostly hematologic abnormalities (51.2%); however, 6 patients did experience immune-related adverse events, though none lead to discontinuation [44••]. Notably, all patients enrolled in this trial had untreated RT, and 71.4% was untreated for their CLL as well, a factor to be considered when comparing ORR and OS to other studies. Keeping in mind the treatment population, these durable responses are promising compared to treatments from traditional CIT regimens.

### Anti-CD20 mAb in Combination with Lenalidomide

A recent phase 1 trial studied the combination of obinutuzumab, high-dose methylprednisolone, and lenalidomide for treatment of patients with RT [45]. Seven patients were treated, and results demonstrated a median PFS of 5 months and median OS of 17 months. ORR was 43% (95% CI, 10–82%) with 2 patients achieving a CR (one patient with DLBCL and one with classical Hodgkin Lymphoma). Results of this study combined findings of a case series and phase 1 clinical trial (closed early due to slow accrual) [45]. Given the potential of lenalidomide to have increased activity in transformed lymphomas compared to de novo DLBCL and to have activity in CLL, combination lenalidomide-based regimens could offer a promising therapeutic option in RT [46, 47].

## Combination Treatment Using PI3K Inhibitors

The PI3K inhibitors (PI3Ki), idelalisib and duvelisib, are approved by the FDA for treatment of R/R CLL [48, 49]. Furthermore, preclinical data using RT patient-derived xenograft (PDX) models demonstrated that in vivo testing of duvelisib combined with venetoclax inhibited tumor growth in two PDX models achieving CR. Results suggest that this combination of treatment produces a synergistic effect such that PI3K inhibition eventually causes degradation of c-Myc and Mcl-1, making cells more sensitive to BCL-2 inhibition [50]. In one phase 1/2 study, eight patients with RT were treated with venetoclax and duvelisib; three patients achieved CR and 1 achieved PR [51]. Similarly to the BTKi, combination PI3Ki and PD-1 inhibition is also being explored. In initial results from a phase 1 study of PI3Ki, copanlisib, in combination with nivolumab for treatment of RT, demonstrated an ORR of 27% in 13 total patients [52]. Lastly, a phase 1 study of TG1501 (anti PD-L1) with umbralisib and ublituximab for CLL or RT (NCT02535286) has been completed, but results are yet to be reported. It is too early to tell whether PI3K inhibition has a role in the treatment of RT.

## Bispecific T Cell Engagers

Blinatumomab, an anti-CD-19/CD3 bispecific antibody (BsAb), is approved for treatment of R/R B-ALL. It has also been studied in R/R DLBCL, with a 43% ORR observed [53]. The multicenter, phase 2 trial BLINART studied blinatumomab in patients with RT who failed to achieve a CR after R-CHOP [54, 55]. Out of all the patients who received R-CHOP (*n* = 34), 24 went on to receive blinatumomab induction, of which 20% achieved a CR, 16% a PR, and 24% had stable disease. Forty-six percent of all patients had an overall response and 36% achieved CR, as compared to 29% with CR after R-CHOP only, suggesting that blinatumomab does improve response after R-CHOP in patients with RT [54, 55].

Epcoritamab, an anti-CD20/CD3 BsAb, was studied in the phase 1b/2 trial—EPCORE-CLL1 [56]. Early results from this study demonstrate an ORR of 60% and CR rate of 50% in 10 patients with RT. A known toxicity of both CART and BsAb, cytokine release syndrome (CRS), occurred in 90% of patients (40% grade 1, 50% grade 2), but no patients discontinued treatment due to CRS. Final results and long-term follow-up data are pending; however, initial results appear to suggest a robust and rapid treatment response with the majority of responses noted at the six-week assessment [55, 56]. Given the promising long-term follow-up results for all patients with large B-cell lymphomas in the EPCORE-NHL1 trial, epcoritamab has been approved for treatment of R/R non-Hodgkin lymphoma (NHL) [57].

Glofitamab, another anti-CD20/CD3 BsAb, has shown promising results in R/R B-cell lymphomas and in RT [58]. Eleven patients with RT received glofitamab for 12 cycles, and results demonstrated an ORR 63.6% [59••]. CR rate was 45.5%, and median time to CR was 3 months. When CR was achieved, response was durable with 80% of patients with ongoing response at ≥ 24.9 months. CRS was a common side effect and occurred in 72.7% of patients despite pretreatment with obinutuzumab a week before the first dose [59••].

Other BsAb being studied in B cell lymphomas include mosunetuzumab (NCT05207670) and TG1801 (NCT04806035). Given the impressive results observed using BsAb for DLBCL, these agents may have potential to treat patients with RT, and epcoritamab currently can be utilized for R/R RT.

## CAR T Cell Therapy

CART has revolutionized the treatment of R/R DLBCL [57, 60, 61]. Given remarkable responses with CART observed in this difficult to treat disease, this therapy has also been used for the treatment of RT. We published our experience treating 9 patients with the anti-CD19 CART, axicabtagene ciloleucel, for RT [62]. In this study, 8 patients responded to therapy, with one patient death resulting from complications of therapy [62]. Clinical trial data evaluating CART for RT is even more limited. Preliminary results of a prospective clinical trial in Israel reported on 8 patients who received anti-CD19 CART for RT. Of these 8 patients, 5 patients had a CR and 3 had progressive disease [63]. In another recently published clinical trial, authors report the results of 6 patients treated with a novel anti-CD19 CART. Of these 6 patients, 5 were infused, 4 had a response, and 3 patients had a CR [64]. In trials that led to the approval of CART, patients with RT were typically excluded from enrollment. However, in the phase 3 TRANSCEND study, 6 patients with RT were included, but outcomes have not been reported [65]. Additionally, 5 patients with RT were included in the trial of a CART developed at Fred Hutchinson Cancer Center (JCAR014), of which 2 patients attained a CR and 1 patient a PR [66].

Given these findings, there are now three ongoing trials that specifically utilize CART for RS. We recently opened the phase 2 investigator-initiated trial, NCT05873712, which combines lisocabtagene maraleucel (liso-cel) with zanubrutinib for the treatment of RT. Patients must have R/R RT, defined as developing RT while on therapy, or R/R disease after 1 prior line of therapy. Another interesting trial combines ibrutinib, Liso-cel, and nivolumab (NCT05672173) and is currently enrolling at City of Hope. Lastly, there is an industry sponsored study prospectively evaluating brexucabtagene autoleucel for rare B-cell malignancies, including RT (NCT05537766). Although current data is from studies with few patients and only short follow-up, results support the use of CART therapy for patients with RT, especially if they are not fit for allogeneic stem cell transplantation.

## Hematopoietic Stem Cell Transplantation

Due to the limited efficacy of CIT and poor OS seen with RT, allogeneic hematopoietic stem cell transplantation (allo-HCT) is offered as consolidative treatment in RT. Allo-HCT, used in the consolidative setting of DLBCL-RT, provides durable responses with one study resulting in a median post-transplant survival of 55.4 months; however, most patients with RT are not candidates for transplantation due to co-morbidities or chemotherapy-resistant disease [2, 67]. Allo-HCT is best utilized in chemotherapy-sensitive RT as evidenced by superior OS compared to those who underwent stem cell transplantation in the setting of progressive disease [67].

Currently, the American Society for Transplantation and Cellular Therapy recommends allo-HCT as front-line consolidation for RT after achievement of objective response to anthracycline-based chemotherapy [68]. In a previous systemic review/meta-analysis, allo-HCT used in the treatment of RT was found to yield an ORR of 79% [69]. Similarly, Kim et al. found favorable outcomes in allo-HCT in RT with an extended four-year OS of 53% [70]. In a study using the Center for International Blood and Marrow Transplant Research (CIBMTR) registry data, the use and outcomes of autologous hematopoietic stem cell transplantation (auto-HCT) and allo-HCT in DLBCL-RT were reviewed [71]. Allo-HCT and auto-HCT in RT resulted in durable remissions despite high-risk features, and a deeper response at the time of transplantation was associated with improved outcomes (3-year PFS/OS 66%/77% CR vs 43%/57% PR vs 5%/15% resistant, *P* < 0.0001) as previously observed in other studies [67, 71]. Taken together, we recommend utilizing allo-HCT in patients with RT who are fit and have good disease control.

## Other Novel-Targeted Agents and Upcoming Trials

Given advancements in drug development, there are various other trials currently underway for the treatment of RT. The WAVELINE-001 study is a phase 1 trial of antibody drug conjugate, zilovertamab vedotin [72]. This drug targets receptor tyrosine kinase-like orphan receptor 1 (ROR1), a cell surface protein expressed on de-differentiated cancer cells, but absent in mature tissues. This study enrolled seven patients with RT. ORR was 57% in the RT cohort with 3 patients achieving CR and 1 patient achieving PR. Median PFS and OS were 4.7 and 24 months respectively [72].

DTRM-555 is a drug consisting of BTKi DTRMWXHS-12, everolimus, and pomalidomide which is being studied in patients with R/R lymphomas [73]. The hypothesis behind this approach is to use BTK and mTOR inhibition coupled with an IMiD to target multiple signaling pathways to improve apoptosis and reduce drug resistance. This drug was well tolerated in patients with R/R lymphomas with majority of adverse events reported as grades 1–2, without any patients needing to discontinue due to toxicities. Results demonstrate that out of 10 evaluable patients, all achieved a PR with 5 out of 10 continuing therapy [73]. Currently, there is an ongoing trial to study DTRM-555 in R/R CLL and R/R NHL. There will be 5 specific disease cohorts with one dedicated to patients with RT (NCT04305444).

NX 1607 is a first in class Casitas B-lineage lymphoma proto-oncogene B (CBL-B) inhibitor which is being studied in a variety of solid tumor malignancies as well as DLBCL and RT (NCT05107674). CBL-B is an E3 ubiquitin ligase which acts as a regulator of T and NK cell activation. Inhibition of CBL-B is thought to restore response in exhausted T cells and increase cytokine production in NK cells [74]. Phase 1 studies will hopefully elucidate whether this novel category of drugs will be a promising immune-oncology target.

PRT2527 is a CDK9 inhibitor that has shown promising antileukemic activity in ALL and CLL in preclinical models [75]. CDK9 is known to be upregulated in solid tumor and hematologic malignancies and, as a result, thought to be a promising therapeutic target [76]. A phase 1 dose escalation trial of PRT2527 for treatment of R/R hematologic malignancies including RT is currently open and recruiting (NCT05665530). Another CDK9 inhibitor VIP152 is also being evaluated in a phase 1 clinical trial for treatment of R/R CLL or RT (NCT04978779).

BCL2/BCLX inhibitor, LP-118, was studied in preclinical models and demonstrated potent activity in venetoclax resistant patient samples and cell lines. A phase 1 trial is currently ongoing evaluating the safety of LP-118 in a variety of B cell malignancies including RT (NCT04771572).

## Conclusion

In summary, patients who develop RT in the context of underlying CLL or at initial presentation require prompt workup and initiation of treatment. Currently, clinical trial enrollment remains a first-line recommendation for patients due to the lack of durable responses observed with traditional CIT regimens.

In this review, we have outlined a variety of regimens which combine standard CIT regimens with targeted agents with the goal of creating a synergistic effect in controlling disease. In addition, recently completed trials highlight a variety of novel agents which may have potential utility in the treatment of RT. Given the current treatment landscape, allo-HCT is one of the only options available for fit patients to achieve a long-lasting remission—a result often dependent on a deep treatment response prior to transplant. Perhaps the emergence of new combinations of treatments and development of novel targeted agents will not only allow for more efficacious bridging treatments for patients prior to allo-HCT, but also offer effective long-term disease control in patients who are transplant ineligible. Given historical poor OS seen with RT, we hope that the breadth of the trials currently available for patients with RT may bring exciting new treatment options for patients in clear need of improved therapies.

## Data Availability

Not applicable.
